# Frailty in Very Elderly Patients is Not Associated with Adverse Surgical or Oncological Outcomes in Extremity Surgery for Soft Tissue Sarcoma

**DOI:** 10.1245/s10434-021-11292-4

**Published:** 2022-02-03

**Authors:** Gausihi Sivarajah, Emma Davies, Anna Hurley, Dirk C. Strauss, Myles J. F. Smith, Andrew J. Hayes

**Affiliations:** 1 Department of Surgery, The Royal Marsden NHS Foundation Trust, London, UK; 2 Institute of Cancer Research, 237 Fulham Road, London, SW3 6JB UK

## Abstract

**Background:**

While surgery remains the mainstay of treatment for limb sarcoma, extreme old age is a relative contraindication to oncological surgery.

**Methods:**

Patients >80 years referred with primary extremity soft-tissue sarcoma (ESTS) between 2007 and 2016 were retrospectively reviewed. Prognostic variables, including ASA status and Clinical Frailty Scores, were collected. Endpoints were perioperative morbidity, locoregional (LRR) and distant recurrence (DR), disease-specific survival (DSS) adjusted using competing risk modelling, and overall survival (OS).

**Results:**

A total of 141 primary tumours were identified, with 116 undergoing resections. Main motives for nonoperative management were severe frailty or significant comorbidity (56.0%). The operative group had a median age of 84 (range 80-96) years and median follow-up of 16 months (range 0-95). 45.7% of patients received radiotherapy. Median hospital stay was 7 (range 0-40) days, with frailty (*p* = 0.25) and ASA (*p* = 0.28) not associated with prolonged admission. 12.9% developed significant complications, with one perioperative mortality.

24.1% had LRR, occurring at a median of 14.5 months. All patients with reported DR (28.4%), except one, died of their disease. Frailty did not confer a significant difference in adjusted LRFS (*p* = 0.95) and DMFS (*p* = 0.84). One- and 5-year adjusted DSS and OS was 87.0% versus 74.9% and 62.3% versus 27.4%, respectively. Frailty (CFS ≥4) was associated with worse OS (hazard ratio [HR] 2.49; 95% confidence interval [CI] 1.51-4.12; *p* < 0.001), however not with adjusted DSS (*p* = 0.16). Nonoperative management conferred a 1- and 5-year adjusted DSS was 58.3% and 44.4%, respectively.

**Conclusions:**

Extremity surgery for sarcoma is well tolerated in the frail very elderly population with low morbidity and comparable oncological outcomes.

Advances in healthcare have resulted in an aging population.^1^ Consequently, an increasing number of older people undergo elective surgery, in which there is a frailty prevalence of 40–50%.^2,3^ Advanced age and frailty has been previously associated with poor perioperative outcomes and considered an independent risk factor for prolonged hospital stay and institutional discharge.^3–5^ Recent studies, however, show similar perioperative and oncological outcomes in older patients (>65 years) compared with those younger, particularly if they are preassessed appropriately.^3,6,7^

Rather than simply relying on age as a predictor of surgical outcome, focus has moved to frailty as a potentially more reliable predictor for perioperative morbidity and mortality. There is no universal definition of frailty, but it is recognised as a decrease in physiological reserve and decreased resistance to stressors. Although frailty is more prevalent in the elderly, the two terms are not synonymous. This is key, as despite chronological ages having similar surgical outcomes, there is still evidence that potentially curative surgical treatment is not being offered, yet desired, in the very elderly population (here defined as those aged 80 years and older).^6,7^

To our knowledge, there is limited literature assessing outcomes of primary extremity soft tissue sarcoma (ESTS) according to frailty following surgery, but our clinical impression was that surgery for sarcomas arising on the extremities was much better tolerated than for intra-abdominal or retroperitoneal sarcomas, in the extreme older age population. In our specialist institution, ESTS surgery is routinely considered for patients older than 80 years. The goal of this study was to determine whether extreme age or frailty results in poorer perioperative and oncological outcomes.

## Methods

### Patient Selection

All patients older than aged 80 years who were referred for consideration of surgical treatment of a primary extremity sarcoma were identified from a single institution over a 10-year period (January 2007 to December 2016). Extremity soft tissue sarcoma (ESTS) was defined as any histologically proven soft tissue sarcoma arising distally from the upper or lower limb girdle, i.e., including the buttock, groin, and shoulder girdle, but excluding the truncal wall. Patients who underwent surgical treatment and those who had nonoperative management were included in the study population. The operated cohort included patients undergoing resectional surgery and regional chemotherapy by isolated limb perfusion. The nonoperated cohort included patients who were treated either by radiotherapy or chemotherapy and those patients who were offered symptomatic treatment only.

Relevant clinicopathological variables were included: histological subtypes, tumor grade, maximum tumor diameter, tumor depth, margin status, functional status, and frailty score. Tumor grade was determined by using French Federation of Cancer Centres Sarcoma Group Grading System (FNCLCC).^8^ The World Health Organisation (WHO) Classification of Soft Tissue Sarcoma was used for sarcoma histotype classification.^9^ Tumor depth was defined as superficial or deep depending on anatomical relationship to deep fascia. Margin status was determined histopathologically as R0 (complete macroscopic resection and microscopically negative margins) and R1 (complete macroscopic resection but microscopically positive margins), and R2 (residual macroscopic tumor as determined by operating surgeon following resection). Comorbid status was measured by the ASA (American Society of Anaesthesiologists) Physical Status Classification System. There is lack of consensus on a single clinical definition of frailty, and therefore we used the most prevalent and widely recognised Rockwood Clinical Frailty score, using a score of ≥4 to distinguish clinical frailty. Postoperative morbidity and mortality were classified according to the Clavien-Dindo scale (III-IV).

### Statistical Analysis

Descriptive data are shown as median (range). Endpoints were overall survival (OS), disease-specific survival (DSS), local recurrence-free survival (LRFS), and distant metastasis-free survival (DMFS) and were calculated from time of surgery to event. Overall survival and DSS probabilities were estimated by using the Kaplan-Meier method and compared with the log-rank test. Cumulative incidence function (CIF) was used to plot sarcoma-related deaths (SRD), adjusting for other-cause deaths, using competing risk methodology.

Univariate and multivariate analysis was performed with Cox proportional hazard model for OS and DSS, and Fine-Gray subdistribution hazard model for sarcoma-related deaths (inversely adjusted DSS). Variables with a univariate *p* value <0.25 level were included in the multivariate analysis after using stepwise methods. Hazard ratios are reported with 95% confidence interval and statistical significance at *p* < 0.05. Statistical analyses were performed in STATA version 15.0 (StataCorp. 2017. *Stata Statistical Software: Release 15*. College Station, TX: StataCorp LLC) with stcompet and stcrprep packages used for competing risk regression.^10,11^

## Results

Between January 2007 and December 2016, a total of 141 patients older than 80 years were referred with primary extremity soft tissue tumours. Patient demographics and clinicopathological variables are included in Table 1.

**Table 1:** Comparison of patient demographics, tumour characteristics and treatment of patients

	Operative group				Nonoperative**	
Variable	CFS 1-3	CFS ≥ 4	Overall	*p* value*	Overall	*p* value^a^
No. patients	56 (48.3)	60 (51.7)	116 (100)		25 (100)	
Gender (Male : Female)	31:25	31:29	62:54	0.69	16:9	0.34
Age	83 (80-95)	85 (80-96)	84 (80-96)	0.39	85 (80-94)	0.28
*ASA*^*b*^						
1	3 (5.36)	0 (0)	3 (2.6)	–	3 (12.0)	–
2	34 (60.7)	22 (36.7)	56 (48.3)	<0.01	10 (40.0)	0.23
3	9 (16.1)	29 (48.3)	38 (32.8)	<0.001	12 (48.0)	0.07
Site of tumour				0.06		<0.05
Upper limb	14 (25.0)	23 (38.3)	37 (31.9)		22 (88.0)	
Lower limb	42 (75.0)	37 (61.7)	79 (68.1)		3 (12.0)	
Maximal tumour size (cm)	9 (1.0-25)	10 (2.5-28.5)	9 (1-28.5)	0.28	13 (5.5-28)	<0.05
*Histological sub-type*						
WD Liposarcoma	4 (7.1)	4 (6.7)	8 (6.9)	0.46	1 (4.0)	0.30
DD/myxoid/pleomorphic liposarcoma	6 (10.7)	4 (6.7)	10 (8.6)	0.22	0 (0)	-
Myxofibrosarcoma	13 (23.2)	8 (13.3)	21 (18.1)	0.08	10 (40.0)	<0.01
Undifferentiated Pleomorphic Sarcoma	22 (39.3)	24 (40.0)	46 (39.7)	0.47	10 (40.0)	0.49
Leiomyosarcoma	2 (3.6)	7 (11.7)	9 (7.8)	0.05	2 (8.0)	0.48
Other	9 (16.1)	13 (21.7)	22 (19.0)	0.22	2 (8.0)	0.09
*Tumor grade*						
1	7 (12.5)	8 (13.3)	15 (12.9)	0.45	5 (20.0)	0.18
2	22 (39.3)	17 (28.3)	39 (33.6)	0.11	15 (60.0)	<0.01
3	27 (48.2)	35 (58.3)	62 (53.4)	0.14	5 (20.0)	<0.005
*Type of operation*						
Resection	55 (98.2)	56 (93.3)	111 (95.7)	0.10	–	–
Amputation	1 (1.8)	3 (5.0)	4 (3.4)	0.17	–	–
Isolated limb perfusion, with subsequent resection	0 (0)	1 (1.7)	1 (0.9)	–	–	–
Local/regional anesthetic	0 (0)	10 (16.6)	10 (8.6)	–	–	–
Treatment other than surgery				0.29		–
Neoadjuvant radiotherapy	10 (17.9)	7 (11.9)	17 (14.7)		2 (8.0) ^c^	–
Adjuvant radiotherapy	20 (35.7)	16 (27.1)	36 (31.0)		–	
Palliative radiotherapy	–	–	–		13 (52.0)	
Palliative chemotherapy	–	–	–		1 (4.0)	

Cells reporting patient characteristics contain either n (column %) for dichotomous variables or median (min-max range) for continuous variables

*WD* well-differentiated, *DD* de-differentiated

*p value (*χ*^2^) for differences between by Clinical Frailty Scores in the operative group

**Median CFS x

^a^*p* value (*χ*^2^) for overall differences between operative and non-operative groups of patients

^b^19 missing values for operative group

^c^RT given with intention to proceed to surgery

### Operative Patient Cohort

A total of 116 tumors were resected in 115 patients. The median age at operation was 84 (range 80-96) years, and 53.4% were males. The median length of follow-up was 16 (range 0-95) months. The median maximal tumour size was 9 cm, and 87.1% were high grade (2-3).

Of the 116 resections, 111 underwent tumour excisions, 4 required amputations, and 1 patient underwent isolated limb perfusion (ILP) followed by resection. The majority presented in the lower limb (68.1%) with the remaining in the upper limb. Twenty-two patients required plastic surgery reconstruction, ranging from split-skin graft to free flaps, following resection. Local or regional anaesthetic was used in ten patients.

A total of 45.7% of patients received radiotherapy—32.0% as neoadjuvant treatment. However, when excluding patients with atypical lipomatous tumours/well-differentiated liposarcomas, 49% of the patients with remaining histotypes received radiotherapy. Frailty (CFS ≥4) (*p* = 0.12) and comorbidity (*p* = 0.84) were not found to be associated with whether radiotherapy was given. Having radiotherapy was not associated with LRFS (*p* = 0.68) or DMFS (*p* = 0.27), even when histotype was considered. One patient had small-volume pulmonary metastases identified before surgery; however, no patients received chemotherapy.

The median hospital length of stay was 7 (range 0–40) days. One-way analysis of variance showed that frailty and increasing ASA status were not associated with prolonged admission (*p* = 0.26 and *p* = 0.28 respectively). 85.3% of patients returned to their place of residence on discharge, with a further 4.3% to a relative’s home. Referral for initial or additional community services were made for 9.6% of these patients.

Fifteen patients developed complications (Clavien-Dindo III-IV); 13 required further operative treatment: 3 for injury-related wound dehiscence (such as after a fall), 5 for washout due to infection, 2 for hematoma evacuation, and 3 for involved margins. One patient required CCU admission for respiratory support for pneumonia, and one patient died following induction of general anesthesia in a patient with ASA 3 and CFS 4. There was no significant association between perioperative morbidity and frailty or ASA status, or whether radiotherapy was given (*p* = 0.61, *p* = 0.67, and *p* = 0.30, respectively).

For the cohort, 28 (24.1%) patients locally recurred at a median time of 14.5 (range 1–48) months. Thirty-three patients (28.4%) developed distant metastases, occurring at a median time of 6.5 (range 0–36) months. Except for the one patient who underwent a pulmonary metastatectomy, all patients with distant recurrence had disease-related mortality. One patient had suspected subcentimeter lung metastases on presentation, however, was offered surgery due to their primary limb sarcoma being symptomatic. Overall, frailty did not confer a significant difference in adjusted LRFS (*p* = 0.95) or DMFS (*p* = 0.84).

**Fig. 1:**
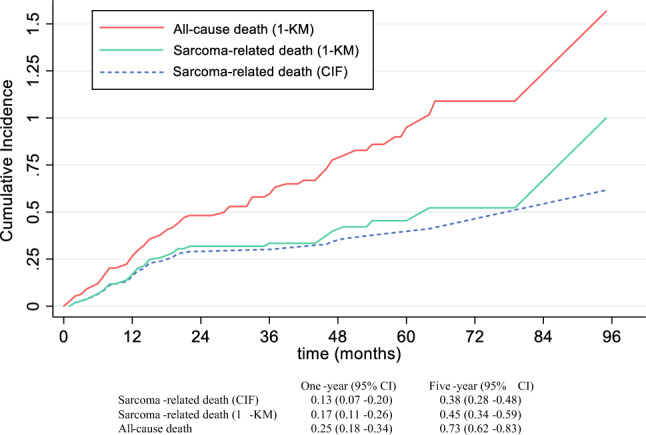
Cumulative incidence functions and Kaplan-Meier failure estimates in operative patient cohorts with primary ESTS. *CIF* cumulative incidence function; *KM* Kaplan-Meier

**Fig. 2:**
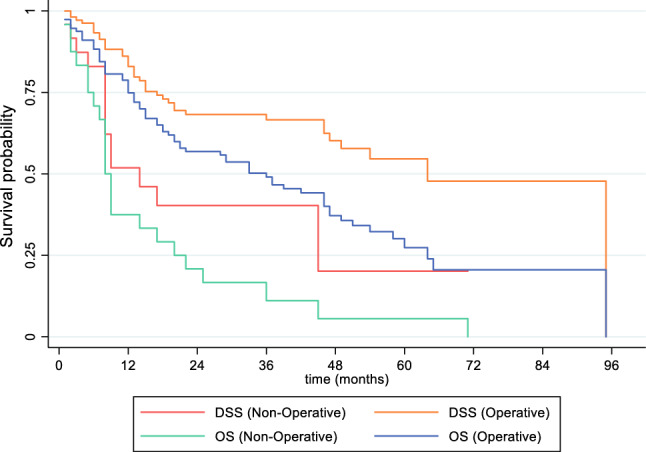
Kaplan-Meier curves for overall survival (*p* < 0.001) and disease-specific survival (*p* = 0.002) comparing operative treatment with nonoperative management for patients with primary ESTS

**Fig. 3:**
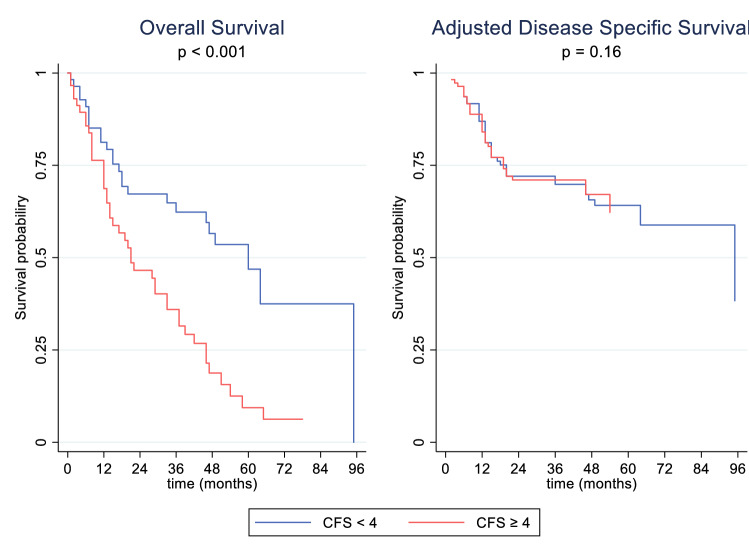
Kaplan-Meier survival curves of patients who underwent surgery, by Clinical Frailty Score

At the end of the study period, 69 operative patients had died: 38 sarcoma-related deaths and 31 from other causes. Disease-specific survival at 1 year and 5 years was 83.0% and 54.6%, respectively. When death from other causes was considered as a competing risk, sarcoma-specific survival at 1 year and 5 years was 87.0% and 62.3%, respectively. Overall survival at 1 year was 74.9% and at 5 years was 27.4% (Figs. 1 and 2). Only increasing tumor size, histopathological grade, and depth were associated with worse DSS and OS on univariate analysis (Table 2). Frailty (CFS ≥ 4) was associated with worse OS HR 2.49 (95% CI 1.51-2.49) *p* <0.001, however, was not associated with DSS (cause-specific HR 1.89, 95% CI 0.98-3.68; *p* = 0.06; subdistribution HR 1.57, 95% CI 0.84-2.93; *p* = 0.16) (Fig. 3).

**Table 2:** Univariate analysis of prognostic factors of oncological outcome on patients who underwent surgery

	Overall survival		Disease-specific survival		Adjusted DSS	
	HR**	*p* value	HR**	*p* value	Subdistribution HR^b^	*p* value
*Gender*						
Male	1.00 (reference)		1.00 (reference)		1.00 (reference)	
Female	0.75 (0.46-1.22)	0.24	0.59 (0.30-1.16)	0.12	0.56 (0.29-1.09)	0.09
*Age (yr)*						
80-84	1.00 (reference)		1.00 (reference)		1.00 (reference)	
85-89	1.42 (0.86-2.35)	0.17	1.20 (0.62-2.35)	0.59	1.10 (0.56-2.14)	0.78
≥90	0.84 (0.37-1.90)	0.67	0.39 (0.09-1.68)	0.21	0.39 (0.11-1.40)	0.15
*Clinical frailty score*						
<4	1.00 (reference)		1.00 (reference)		1.00 (reference)	
≥4	2.49 (1.51-4.12)	<0.001	1.89 (0.98-3.68)	0.06	1.57 (0.84-2.93)	0.16
*ASA*						
1	1.00 (reference)		1.00 (reference)		1.00 (reference)	
2	2.67 (0.36-19.8)	0.34	1.40 (0.18-10.6)	0.75	1.10 (0.16-7.82)	0.94
3	5.99 (0.80-45.1)	0.08	2.71 (0.35-21.2)	0.34	1.70 (0.23-12.4)	0.60
*Tumor size (cm)*^*a*^						
≤5.4	1.00 (reference)		1.00 (reference)		1.00 (reference)	
5.5-13.9	2.87 (1.39-5.94)	<0.05	1.93 (0.76-4.87)	0.17	1.66 (0.69-4.01)	0.26
≥14	3.09 (1.40-6.81)	<0.05	2.97 (1.12-7.85)	<0.05	2.40 (0.97-5.97)	0.06
*Tumor grade*						
1	1.00 (reference)		1.00 (reference)		1.00 (reference)	
2	1.65 (0.49-5.51)	0.42	4.63 × 10^8^	–	3.40 × 10^6^	<0.001
3	2.41 (0.74-7.77)	0.14	6.51 × 10^8^	<0.001	4.20 × 10^6^	<0.001
*Tumor depth*						
Superficial	1.00 (reference)		1.00 (reference)		1.00 (reference)	
Deep	2.19 (1.25-3.85)	<0.05	2.77 (1.21-6.33)	<0.05	2.15 (1.01-4.57)	<0.05
*Margins*						
R0	1.00 (reference)		1.00 (reference)		1.00 (reference)	
R1	0.97 (0.60-1.57)	0.90	0.97 (0.50-1.88)	0.92	1.09 (0.58-2.04)	0.80
R2	1.55 (0.37-6.53)	0.55	2.45 (0.56-10.6)	0.23	2.85 (0.8-9.59)	0.09
*Treatment*						
Surgery alone	1.00 (reference)		1.00 (reference)		1.00 (reference)	
Surgery + RT	0.69 (0.42-1.11)	0.13	1.04 (0.54-1.98)	0.91	1.27 (0.68-2.37)	0.45

Values in parentheses are 95% confidence intervals

*HR* Hazard ratio

**Cox proportional hazard model

^a^Maximal tumour size stratified by interquartile range

^b^Fine-Gray subdistribution hazard model

### Nonoperative Management

Within the study period, 25 patients with primary ESTS were identified to have not undergone any surgery. The median age in this cohort at review was 85 (range 80-94) years, with a median maximal tumor size of 13 cm, and 80.0% of tumors were high grade. The main reasons for surgery not being performed (56.0%) was determined by the patient’s severe frailty (CFS ≥ 6) or significant comorbidity (ASA 3). Other exclusions were that the tumor was considered inoperable, patient refusal, and the identification of significant metastatic disease after referral. Palliative treatment (radiotherapy or once weekly low-dose Paclitaxel) was offered to 88.0% of these patients, with 72.7% of this group, undertaking treatment.

In this cohort, the median time from histological diagnosis to death was 8 (range 0-71) months; overall survival at 1 and 5 years was 37.5% and 5.6%, respectively. 56.0% of patients died of their disease; 1- and 5-year DSS were 51.8% and 20.2%, respectively, and when adjusted for competing risk, 58.3% and 44.4%, respectively (Fig. 2). This poorer oncological prognosis in the nonoperative cohort is demonstrated to be significant (OS *p* < 0.001, adjusted DSS *p* < 0.05) compared with patients who underwent surgery. Only one patient remained alive with their disease at the end of observation, with the histological diagnosis of well-differentiated liposarcoma.

## Discussion

### Overall Outcomes

The world population is aging, and consequently more elderly patients are presenting for surgery. Extreme age and frailty have been associated in the literature with adverse health events following surgery and poor perioperative outcomes.^12–14^ This retrospective study demonstrates that even in a cohort of patients at the extremes of age and with significant clinical frailty, resectional surgery can be associated with good perioperative outcomes, despite similar clinicopathological variables that may be seen in a younger cohort.^13^

### Tumor Characteristics

Previous studies have contributed to the knowledge that increasing age is associated with worse LRFS and DSS,^15–17^ reflected by elderly patients having significantly high-grade tumors, more aggressive histology, and a different distribution of sarcoma histotypes.^18–22^ Tumor prognostic factors captured in this study—large tumor size, high grade, and intramuscular tumors—are significant independent adverse prognostic factors for sarcoma specific survival, which parallels the literature. Furthermore, after excluding confounding patient-related variables (age, frailty, and comorbidities), tumor grade is still seen as associated with sarcoma-related prognosis (*p* < 0.001) (Table 3).

**Table 3:** Multivariable analysis of prognostic factors of oncological outcome on patients who underwent surgery

	Overall survival		Disease-specific survival		Adjusted DSS	
	HR**	*p* value	HR**	*p* value	Sub-distribution HR^b^	*p* value
*Gender*						
Male	1.00 (reference)		1.00 (reference)		1.00 (reference)	
Female	*	*	*	*	0.64 (0.32-1.27)	0.20
*Age (yr)*						
80-84	1.00 (reference)		1.00 (reference)		1.00 (reference)	
85-89	*	*	*	*	*	*
≥90	*	*	*	*	*	*
Clinical frailty score						
<4	1.00 (reference)		1.00 (reference)		1.00 (reference)	
≥4	1.80 (0.93-3.51)	0.08	*	*	*	*
*ASA*						
1	1.00 (reference)		1.00 (reference)		1.00 (reference)	
2	2.88 (0.35-23.6)	0.32	2.32 (0.28-19.2)	0.44	*	*
3	4.85 (0.53-44.1)	0.16	5.71 (0.63-51.4)	0.12	*	*
*Tumor size (cm)*^*a*^						
≤5.4	1.00 (reference)		1.00 (reference)		1.00 (reference)	
5.5-13.9	2.11 (0.88-5.08)	0.09	1.21 (0.44-3.36)	0.72	*	*
≥14	3.77 (1.31-10.8)	<0.05	3.22 (0.95-10.88)	0.06	*	*
*Tumor grade*						
1	1.00 (reference)		1.00 (reference)		1.00 (reference)	
2	2.49 (0.70-8.89)	0.16	6.11 × 10^8^	–	4.13 × 10^8^	–
3	3.52 (0.97-12.7)	0.06	9.01 ×10^8^	<0.001	5.37 × 10^8^	<0.001
*Tumor depth*						
Superficial	1.00 (reference)		1.00 (reference)		1.00 (reference)	
Deep	1.96 (0.96-3.99)	0.07	2.74 (1.00-7.50)	0.05	1.95 (0.88-4.29)	0.10
*Margins*						
R0	1.00 (reference)		1.00 (reference)		1.00 (reference)	
R1	*	*	*	*	*	*
R2	*	*	*	*	*	*
*Treatment*						
Surgery alone	1.00 (reference)		1.00 (reference)		1.00 (reference)	
Surgery + RT	0.54 (0.29-1.00)	0.05	*	*	*	*

Values in parentheses are 95% confidence intervals

*HR* Hazard ratio

*Variable excluded on stepwise selection

**Cox proportional hazard model

^a^Maximal tumor size stratified by interquartile range

^b^Fine-Gray subdistribution hazard model

### Patient Management

Multiple studies suggest undertreating of the elderly cohort from both a surgical and radiotherapy arm is common.^23–25^ While this study did not demonstrate an association with local recurrence-free survival, it is known that omitting RT in patients with limb-sparing surgery increases the risk of local recurrence.^26^ The use of a nomogram estimating the risk of local recurrence without radiation may assist in the decision-making but does not take into account patient frailty and comorbid status.^16^ RT has been discussed in the literature and the notion that elderly are poorly tolerant appears to be a widespread misconception.^21,27,28^ The use of hypofractionated radiation as adjuvant and palliative treatment in the elderly has proved to be effective and well-tolerated^29^; however, clinician experience, well-clear surgical margins, consideration of risk of late toxicities against comorbidity, and patients concerns regarding distance and multiple hospital attendances may influence the decision toward omitting radiotherapy.

Adjuvant chemotherapy in the setting of primary ESTS in all adults is still conflicting, although recent studies suggest an improvement on overall survival in high-risk tumors.^30,31^ However, other than a few certain specific sarcoma subtypes, adjuvant chemotherapy is not offered routinely to adults with primary localised STS even in younger populations in U.K. practice, and accordingly chemotherapy was not administered in the elderly population, especially those in whom the incidence of frailty was high, because the potential for treatment-related morbidity is deemed to outweigh oncological benefit. ILP, however, has been demonstrated to be well-tolerated in the advanced age population.^32^

The majority of cases were limb-conserving operations, with only 3.4% of patients undergoing amputations. Amputations in patients with extremity sarcomas are only considered for locally advanced multicompartmental disease, not amenable to limb preservation. In elderly patients, amputation is even more likely to impact on quality of life and require additional community services and rehabilitation. Almost one fifth of the elderly population had surgery with plastics reconstruction, which often are higher risk. This again highlights the ability to perform major surgery in this older population. The use of local and regional anaesthetic in the elderly population assists in avoiding general anaesthetic in the event of considerable comorbidities.

### Oncological Outcomes

Nomograms consider increasing age to be associated with worse overall survival^33,34^; however it is acknowledged that competing unrelated concurrent morbidity has not been taken into account and age is not associated with DMFS. In this elderly population (>80 years), age alone was not found to be an independent prognostic factor for overall survival or for disease-free survival. This reflects multiple new studies also showing no different oncological outcome solely due to age.^3,5,6^ When considering frailty (CFS ≥ 4), there was a significant difference in overall survival but not disease-free survival. This is expected given the poor physiological reserve and increased comorbidities in this cohort.

### Frailty as a Guide and Further Management

Because there is no universal definition or scale to describe frailty, we considered the most widely used scale that is one of the few scores that has demonstrated reliability and validity.^35^ Given this has its limitations, we also included the ASA physical status classification system. While hazard ratios are not statistically significant, there is a trend of increasing ASA status with decreasing overall and disease-specific survival, and a selective bias of patients with significant frailty (CFS ≥ 6) and comorbidity (ASA 3) not undergoing surgery. This suggests poor physiological reserve to be a more prevalent risk factor for worse outcomes rather than chronological age. This has been echoed in other studies showing increased perioperative morbidity due to multiple comorbidities but not chronological age.^22^ While there were patients with CFS 7 (severely frail—dependent for personal care) that did undergo and tolerate surgery in this cohort, for patients classified as CFS 8 (very severely frail—completely dependent and approaching end of life), and CFS 9 (terminally ill—life expectancy <6 months), surgery was considered futile and thus severe frailty is considered a contraindication to surgery.^36^

There are limitations in our study, including the inherent shortcomings of its retrospective design. First, the follow-up period in this series was relatively short with a median follow-up of 16 months. It is known that clinical follow-up often is shorter in elderly populations.^24^ In a centralized tertiary sarcoma service, which often is not local to patients and therefore less accessible, elderly patients are more likely to be discharged to local follow-up. Second, often routine chest radiographs are omitted due to patient choice and the futility of further treatment, resulting in an underreporting of distant recurrent disease in this series. Further to this, the detection of significant pulmonary metastases before surgery lends to a selection bias in not offering surgical treatment to patients for the primary limb tumour. Third, while independence and quality of life status following surgery was determined by reviewing admission and discharge destinations, long-term residence and frailty status was not established. The literature does suggest that elderly patients undergoing oncology surgery, greatly remain at their premorbid functional capacity at 1 year; however, there may be an impact on cognition and performance status.^37,38^ A prospective study would allow for a comprehensive preoperative frailty assessment to predict perioperative recovery and long-term functional outcomes.

## Conclusions

Even at the extremes of elderly age (>80 years), surgery for limb sarcomas is well-tolerated and safe, with low complications rates, and age alone should not be a contraindication for surgery. Frailty was not prognostic for morbidity and oncological outcomes but should still be considered when planning surgery in the elderly population.
